# The History of Epidemic Influenza

**Published:** 1918-10-26

**Authors:** 


### THE HISTORY OF EPIDEMIC INFLUENZA.

Special interest attaches at the present time to the history of previous epidemics of influenza in view of the serious pandemic prevailing. The history of influenza is supposed to date from a very remote period, although authentic information regarding its earlier appearance in Europe and in this country is not available. The first epidemic of a catarrhal fever similar to influenza was recorded in the year 1173. Subsequently, similar epidemics, varying in number from four to eight per century, occurred during the fourteenth, fifteenth, sixteenth, seventeenth, and eighteenth centuries. Between the years 1800 and 1850 there were ten epidemics of influenza. A widespread epidemic which invaded many countries occurred during the years 1830-33. In 1837 there was a serious epidemic in this country, half of the population of London being attacked. Ten years later a particularly severe type of influenza, somewhat similar to the present type, made its appearance, and there were many deaths from respiratory and cardiac complications. At this stage the influenza virus appeared to have exhausted itself, or the resistance of the people had been so increased by previous attacks that they had become more or less immune, for no serious epidemic occurred in this country for forty years, and the existence of such a disease had come to be entirely forgotten except by the older inhabitants. But the year 1890 brought a sudden awakening. In May 1889 influenza appeared in Siberia, and by autumn had spread over Russia. The newspapers of that time record the appearance of a curious epidemic disease in Russia, which greatly interfered with the industrial and social activities of that country. At first it was thought that some new mysterious malady had appeared, but it soon became evident that it was our old friend, or, rather, enemy, influenza. It quickly spread through Poland to Central and Southern Europe, and by the end of December it had reached London, from whence it spread to the provinces. The present epidemic, which originally appeared in Spain, bears a close similarity, in its distribution and the rapidity with which it spreads, to the great epidemic of 1889-90, while in type it conforms to the epidemic of 1848. From a study of the history of influenza in the past it will be seen that while we must expect outbreaks of this disease at varying intervals of time, we may anticipate after each epidemic a lengthened period of immunity from attack.

